# Sex differences in tumor characteristics, treatment, and outcomes of gastric and esophageal cancer surgery: nationwide cohort data from the Dutch Upper GI Cancer Audit

**DOI:** 10.1007/s10120-021-01225-1

**Published:** 2021-08-07

**Authors:** Marianne C. Kalff, Anna D. Wagner, Rob H. A. Verhoeven, Valery E. P. P. Lemmens, Hanneke W. M. van Laarhoven, Suzanne S. Gisbertz, Mark I. van Berge Henegouwen, the Dutch Upper GI Cancer Audit group

**Affiliations:** 1 Department of Surgery, Cancer Center Amsterdam, Amsterdam UMC, University of Amsterdam, Amsterdam, the Netherlands; 2 Department of Oncology, Lausanne University Hospital (CHUV), University of Lausanne (UNIL), Lausanne, Switzerland; 3 Department of Research, Netherlands Comprehensive Cancer Organization (IKNL), Utrecht, the Netherlands; 4 Department of Medical Oncology, Cancer Center Amsterdam, Amsterdam UMC, University of Amsterdam, Amsterdam, the Netherlands; 5 Department of Public Health, Erasmus University Medical Centre, Rotterdam, the Netherlands

**Keywords:** Gastric cancer, Esophageal cancer, Gastrectomy, Esophagectomy, Survival

## Abstract

**Background:**

Sex differences in clinicopathological characteristics, treatment, and postoperative outcomes of gastric and esophageal cancer are largely undefined. This study aimed to compare tumor and treatment characteristics and outcomes of gastric and esophageal cancer surgery between male and female patients.

**Methods:**

Patients after elective surgery for primary esophageal (EAC) or gastric adenocarcinoma (GAC) registered in the Dutch Upper GI Cancer Audit between 2011 and 2016 were included. The primary endpoint, 5-year relative survival with relative excess risk (RER), i.e., adjusted for the normal life expectancy, was compared between male and female patients with EAC and GAC.

**Results:**

In total, 4937 patients were included (75% male) with a mean age of 66 years. cT and cN-stages showed a similar distribution in male and female patients. In females, antrum GAC was more frequent (47% vs. 38%, *p* < 0.001). Female patients with EAC less frequently received neo-adjuvant treatment (OR = 0.60, 95% CI 0.38–0.96, *p* = 0.033). For GAC, less postoperative morbidity (33% vs. 38% *p* = 0.017) and less re-interventions (12% vs. 16%, *p* = 0.008) were observed in females, although they had inferior 5-year relative survival (49% vs. 56%, RER = 1.31, 95% CI 1.09–1.58, *p* = 0.004). No differences in relative survival of EAC were observed.

**Conclusions:**

In addition to significant sex differences in tumor location, female patients with esophageal adenocarcinoma less frequently received neo-adjuvant therapy, and female patients with gastric adenocarcinoma had inferior relative survival. Further consideration and exploration of sex differences in surgical treatment and outcomes are necessary to improve tailored treatment and outcomes.

**Supplementary Information:**

The online version contains supplementary material available at 10.1007/s10120-021-01225-1.

## Introduction

The incidence of gastric and esophageal cancer is substantially higher in male patients [1, 2]. For both gastric and esophageal adenocarcinomas, behavioral risk factors, such as obesity or exposure to carcinogens like tobacco smoking, do not entirely explain the sex disparity in incidence seen across multiple populations [3–5], thus strongly suggesting sex differences in susceptibility and/or biology for this type of cancer. In fact, increasing evidence suggests a sexual dimorphism in cancer biology, and sex-biased molecular signatures have been observed across multiple tumor types [6–10].

Perioperative chemotherapy and neo-adjuvant chemoradiotherapy combined with surgical resection are the mainstays of curative treatment for gastric and esophageal cancer, respectively [11, 12]. While the impact of the patients’ sex on the balance between efficacy and toxicity of systemic treatments in oncology has gained more attention in recent years [13, 14], its impact on surgical outcomes has been investigated less frequently. Previous studies have shown that for both gastric and esophageal cancer, male patients more frequently undergo surgery [15, 16]. Furthermore, in gastric cancer patients treated exclusively with gastrectomy, the Dutch D1D2-trial showed a superior survival in female patients after an extended (D2) lymphadenectomy [17]. In addition, the CROSS-trial, evaluating the benefit of neo-adjuvant chemoradiotherapy versus surgery alone for esophageal cancer, portrayed a more pronounced treatment effect in males [18].

As a result of the difference in incidence, only relatively small absolute numbers of female patients are included in many clinical trials concerning gastric and esophageal cancer treatment, impeding any firm conclusions concerning the magnitude of the treatment benefit in female patients. In the context of the limited data, the aim of this study was to examine sex differences in tumor and treatment characteristics, and outcomes of gastric and esophageal cancer surgery in a large nationwide cohort study, to provide ground for further individualization of gastric and esophageal cancer treatment.

## Materials and methods

Data for this study were acquired from the national Dutch Upper GI Cancer Audit (DUCA) database. In the Netherlands, caregivers are obliged to register all patients with gastric and esophageal cancer with intended resection in the DUCA registry. This audit is part of the Dutch Institute for Clinical Auditing (DICA), and was initiated in 2011 with the aim of providing independent information on the quality of care. Validation of case completeness (99.8%) and accuracy (94–100%) has been performed [19, 20]. Data registration for audit purposes is limited to 30 days after surgery, or when extending over 30 days, the duration of the initial hospital stay. To allow research initiatives regarding survival, DUCA-data were linked with data of the Dutch national health care insurance registry (Vektis). As health care insurance is mandatory for all Dutch inhabitants, this registry includes 99% of the Dutch population. Date of death is registered given that insurance ends as the patient dies. The process of matching datasets and subsequent validation has been described recently [21]. Data collected from the combined dataset consisted of baseline patient characteristics, tumor and treatment specifications, and histopathological and postoperative outcomes, and vital status.

This study was approved by the scientific committee of the DUCA. No informed consent, opt-out procedure, or ethical approval was required under Dutch law. This paper complies with the STROBE guidelines for observational cohort studies [22].

### Patients and treatment

All patients with a primary esophageal or gastric adenocarcinoma that underwent a surgical procedure with curative intent between 2011 and 2016 were retrieved from the DUCA dataset. Patients with missing information on sex, and patients that underwent a salvage or non-elective procedure were excluded. All patients underwent surgery with curative intent and remained in the analyses if, due to unforeseen circumstances (e.g., metastatic disease and tumor extent), no surgical resection was performed. The performed surgical procedures were a transthoracic or transhiatal esophagectomy, a total or partial gastrectomy, or no resection (i.e., bypass or surgical exploration). Multimodal treatment regimens consisted of neo-adjuvant or perioperative treatment (combined neo-adjuvant and adjuvant treatment).

### Outcome data and definitions

The primary outcome was 5-year relative survival after gastric and esophageal cancer surgery. Secondary outcomes were differences in tumor characteristics, treatment specifications, short-term morbidity and mortality, and oncological outcomes such as response to neo-adjuvant treatment. Relative survival was defined as the observed overall survival divided by the age, year, and sex-matched expected overall survival of the general Dutch population [23]. Clinical and pathological TNM staging was defined by the eighth TNM staging edition. Survival was calculated as the interval (in months) from the date of surgery to the date of death or last follow-up (Vektis database last follow-up: 1st of September 2017).

### Statistical analysis

For all outcomes investigated, stratification was performed by tumor location in esophageal and gastric adenocarcinoma. Primary and secondary endpoints were subsequently compared between male and female patients. Mann–Whitney *U* or Student’s *t* test for continuous variables, and *χ*^2^ test for categorical variables were used when applicable.

In case of treatment differences between male and female patients, regarding the application of neo-adjuvant or perioperative treatment, transhiatal or transthoracic esophagectomy for esophageal tumors, and partial or total gastrectomy for gastric tumors, additional multivariable logistic regression analysis was performed to assess whether differences remained after adjustment for clinical parameters thought to affect treatment probability (age, ASA-class, the presence of cardiac, pulmonary, vascular or diabetic comorbidities, clinical T and N stage, tumor differentiation, histopathological subtype, tumor location, and year of surgery), resulting in odds ratios (OR) with 95% confidence intervals (95% CI).

As the general life expectancy is known to differ between sexes, relative survival was assessed as the overall survival observed in the gastric and esophageal cancer patient cohort, divided by the expected survival in the general Dutch population matched on age, sex, and year, according to the method of Pohar Perme [23]. To assess the association between sex and risk of death, multivariable relative excess risk (RER) with 95% CI was estimated using the relative survival, adjusted for confounders known to affect survival (age, ASA-score, clinical T and N stage, and tumor sub-location).

As pre-menopausal female sex hormones, i.e., estrogen, are thought to have a protective effect reducing the risk and invasiveness of gastric and esophageal adenocarcinoma, subgroup analyses comparing male patients to female patients ≤ 55 and > 55 years were performed [6, 24–27].

Few missing data were present in clinical variables and therefore handled by complete case analyses. STATA Version 14.2 (StataCorp, College Station, TX, USA) was used to assess relative survival, and SPSS Statistics Version 26.0 (Armonk, NY) was used for further statistical analysis. Two-sided *p* values of less than 0.05 were considered statistically significant.

## Results

### Baseline characteristics

**Fig. 1:**
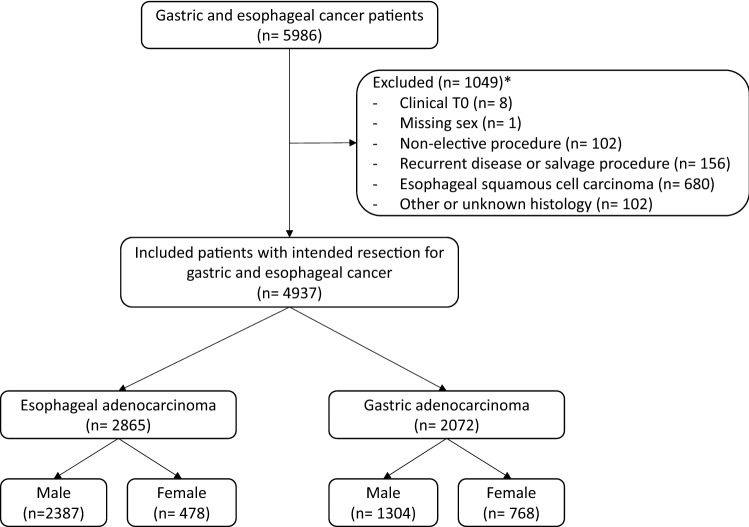
Flowchart of study inclusion. *Multiple reasons for exclusion may apply for one patient

**Fig. 2:**
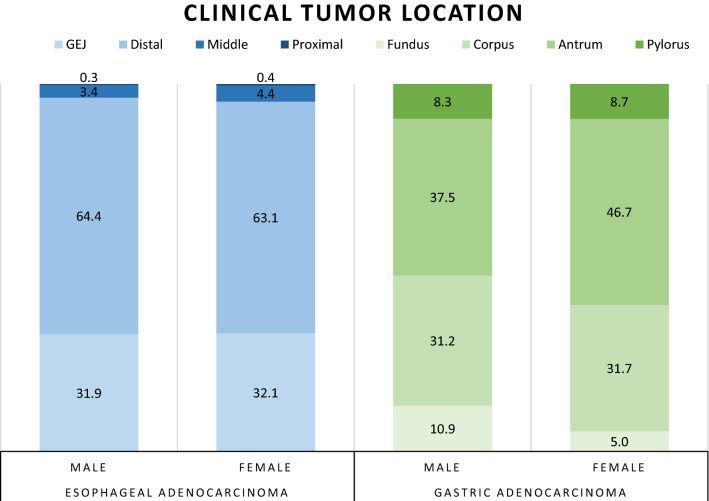
Distribution of tumor location and histology for male and female patients with gastric and esophageal cancer. Numbers represent the percentages of tumor location for male and female patients. *GEJ* gastro-esophageal junction

In total, 2865 patients with esophageal and 2072 patients with gastric adenocarcinoma were included, of whom 74.8% were male (Fig. 1; Table 1). More cardiac comorbidities were observed in male patients in both groups. No sex differences were observed in the distribution of clinical T and N stage. For gastric adenocarcinoma, more poorly differentiated tumors and more diffuse type tumors were observed in females (69.4% vs. 56.5%, *p* < 0.001; 48.6% vs. 34.9%, *p* < 0.001, respectively), who also had tumors more frequently located in the antrum (46.7% vs. 37.5%), while males more often had tumors located in the fundus (10.9% vs. 5.0%, *p* < 0.001; Fig. 2).

**Table 1:** Baseline characteristics of male and female patients with gastric and esophageal cancer

Characteristics		Esophageal cancer					Gastric cancer				
		Male		Female		*p*	Male		Female		*p*
		*n* = 2387		*n* = 478			*n* = 1304		*n* = 768		
		*n*	%	*n*	%		*n*	%	*n*	%	
Mean age	Years (SD)	64.6	9.1	65.0	9.3	0.454	68.6	11.0	67.7	12.9	0.115
Age ≤ 55 year		372	15.6	75	15.7	0.959	158	12.1	146	19.0	< 0.001
Mean BMI	Kg/m^2^ (SD)	26.4	4.0	26.8	5.6	0.114	25.3	4.1	25.1	4.9	0.197
ASA score	I	416	17.6	71	15.1	0.515	170	13.2	123	16.3	0.209
	II	1422	60.1	285	60.5		726	56.2	422	55.8	
	III	519	21.9	112	23.8		385	29.8	206	27.2	
	IV	11	0.5	3	0.6		11	0.9	5	0.7	
Comorbidity	Pulmonary	418	17.5	87	18.2	0.703	244	18.7	91	11.9	< 0.001
	Cardiac	617	25.8	89	18.7	0.001	449	34.4	177	23.1	< 0.001
	Vascular	886	37.1	203	42.6	0.025	522	40.0	283	37.0	0.171
	Diabetes	402	16.8	82	17.2	0.852	217	16.6	123	16.1	0.739
cT stage	Tis	9	0.4	–		0.076	7	0.7	3	0.5	0.363
	T1	115	5.1	37	8.1		76	8.0	57	10.4	
	T2	445	19.6	86	18.8		252	26.3	133	24.2	
	T3	1644	72.2	321	70.2		541	57.0	305	55.6	
	T4	63	2.8	13	2.8		73	7.7	51	9.3	
cN stage	N0	829	36.0	195	42.5	0.115	648	58.1	378	57.6	0.927
	N1	951	41.3	170	37.0		303	27.2	176	26.8	
	N2	427	18.6	75	16.3		108	9.7	63	9.6	
	N3	68	3.0	15	3.3		15	1.3	12	1.8	
	N +	26	1.1	4	0.9		41	3.7	27	4.1	
Differentiation	Good	866	55.2	163	51.7	0.267	437	43.5	178	30.6	< 0.001
	Poor	704	44.8	152	48.3		567	56.5	404	69.4	
Histological subtype	Intestinal	788	81.2	160	82.1	0.562	515	57.7	239	45.0	< 0.001
	Diffuse	116	12.0	19	9.7		311	34.9	258	48.6	
	Mixed	66	6.8	16	8.2		66	7.4	34	6.4	
Clinical tumor location	Cervical	2	0.1	–	–	0.597					
	Proximal	4	0.2	2	0.4						
	Middle	81	3.4	21	4.4						
	Distal	1527	64.4	301	63.1						
	GEJ	757	31.9	153	32.1						
	Fundus						136	10.9	37	5.0	< 0.001
	Corpus						389	31.2	232	31.7	
	Antrum						468	37.5	342	46.7	
Belongs to tumor location	Pylorus						104	8.3	64	8.7	
	Entire stomach						73	5.8	53	7.2	
	Gastric remnant						78	6.3	5	0.7	

Percentages for the variables are calculated out of the total number of actual results available, excluding the missing values. Percentages may not add up to 100% due to rounding

*ASA* American Society of Anesthesiologists, *BMI* body mass index (kg/m^2^), *cN* clinical N stage, *cT* clinical T stage, *GEJ* gastro-esophageal junction, *SD* standard deviation

### Treatment characteristics

Females with esophageal adenocarcinoma less often received neo-adjuvant treatment (78.1% vs. 85.6%; OR 0.60, 95% CI 0.38–0.96, *p* = 0.033; Table 2), which less often consisted of chemoradiotherapy compared to neo-adjuvant treatment regimens in male patients (90.5% vs. 93.9%, *p* = 0.019). Less females with gastric adenocarcinoma received perioperative treatment (35.2% vs. 40.3%, *p* = 0.024), although not significant when adjusted for clinicopathological factors (OR 0.75, 95% CI 0.52–1.06, *p* = 0.105).

**Table 2:** Treatment characteristics of male and female patients with gastric and esophageal cancer

Characteristics		Esophageal cancer					Gastric cancer				
		Male		Female		*p*	Male		Female		*p*
		*n* = 2387		*n* = 478			*n* = 1304		*n* = 768		
		*n*	%	*n*	%		*n*	%	*n*	%	
Neo-adjuvant treatment		2033	85.6	370	78.1	< 0.001	298	23.0	165	21.6	0.463
	Chemotherapy	125	6.1	35	9.5	0.019	275	92.3	159	96.4	0.083
	CRT	1908	93.9	335	90.5		23	7.7	6	3.6	
	> 80% completed	1948	96.8	351	95.9	0.396	200	67.2	110	67.5	0.956
Perioperative treatment^a^		133	5.6	34	7.1	0.188	499	40.3	260	35.2	0.024
Procedure type	Transthoracic	1357	57.0	234	49.3	< 0.001	10	0.8	1	0.1	0.007
	Transhiatal	876	36.8	201	42.3		13	1.0	4	0.5	
	Total gastrectomy	62	2.6	11	2.3		531	41.1	267	35.0	
	Partial gastrectomy	2	0.1	4	0.8		631	48.8	425	55.8	
	No resection^b^	84	3.5	25	5.3		107	8.3	65	8.5	
Approach of surgery	Open	1087	45.6	230	48.1	0.448	932	71.9	528	69.4	0.222
	MIS	1251	52.5	242	50.6		364	28.1	233	30.6	
	Hybrid	44	1.8	6	1.3		NA		NA		
Year of surgery	2011	412	17.3	87	18.2	0.247	193	14.8	103	13.4	0.377
	2012	444	18.6	105	22.0		213	16.3	144	18.8	
	2013	459	19.2	94	19.7		286	21.9	181	23.6	
	2014	514	21.5	99	20.7		342	26.2	181	23.6	
	2015	558	23.4	93	19.5		270	20.7	159	20.7	

Percentages for the variables are calculated out of the total number of actual results available, excluding the missing values. Percentages may not add up to 100% due to rounding

*CRT* chemoradiotherapy, *MIS* minimally invasive surgery, *NA* not applicable

^a^Consisting of both neo-adjuvant and adjuvant treatment

^b^Surgery for intended curative resection, due to metastatic disease, tumor extent, or deterioration of the condition of the patient during surgery, no surgical resection was performed

For both esophageal and gastric adenocarcinoma, the type of surgical procedure performed differed. For esophageal adenocarcinoma, more female patients underwent a transhiatal resection (42.3% vs. 36.8%, *p* < 0.001), and for gastric adenocarcinoma, more female patients underwent a partial gastrectomy (55.8% vs. 48.8%, *p* = 0.007). These differences did not remain significant after adjustment for clinicopathological factors (EAC: OR 0.73, 95% CI 0.50–1.08*, p* = 0.119; GAC: OR 1.23, 95% CI 0.83–1.81, *p* = 0.300). No sex difference was observed for open or minimally invasive approaches.

### Postoperative and histopathological outcomes

A microscopically radical resection (R0) was equally obtained in both sexes (Table 3). Pathological T stage differed for patients with gastric adenocarcinoma, with lower pT stages in female patients (pT3: 37.0% vs. 41.8%, *p* = 0.014). pN stage differed between male and female patients with esophageal adenocarcinoma, while (positive) lymph-node harvest showed no difference. The responses to neo-adjuvant therapy were equally distributed. Only for patients with gastric adenocarcinoma, postoperative morbidity differed; with more complications in general (38.1% vs. 32.9%, *p* = 0.017), more pulmonary complications (15.7 vs. 10.8%, *p* = 0.002), more anastomotic leakages (7.5% vs. 5.1%, *p* = 0.031), and more re-interventions (16.2% vs. 11.9%, *p* = 0.008) in male patients. Postoperative morbidity was higher after total versus partial gastrectomy (41.9% vs. 34.4%, *p* = 0.001). Short-term mortality was comparable between male and female patients in both groups.

**Table 3:** Pathological and postoperative outcomes of male and female patients with gastric and esophageal cancer

Characteristics		Esophageal cancer					Gastric cancer				
		Male		Female		*p*	Male		Female		*p*
		*n* = 2387		*n* = 478			n = 1304		*n* = 768		
		*n*	%	*n*	%		*n*	%	*n*	%	
Lymph nodes, median (IQR)	Number	18	13–24	17	12–24	0.636	18	13–26	19	13–28	0.069
	Positive	0	0–2	0	0–2	0.185	1	0–5	1	0–5	0.717
(y)pT stage	T0	373	16.9	73	16.9	0.815	67	5.7	25	3.6	0.014
	Tis	23	1.0	6	1.4		13	1.1	6	0.9	
	T1	396	17.9	85	19.6		161	13.8	130	18.8	
	T2	450	20.3	79	18.2		184	15.7	117	16.9	
	T3	935	42.3	185	42.7		489	41.8	256	37.0	
	T4	35	1.6	5	1.2		256	21.9	158	22.8	
(y)pN stage	N0	1322	57.7	280	62.2	0.005	538	45.4	320	46.1	0.486
	N1	474	20.7	64	14.2		218	18.4	122	17.6	
	N2	307	13.4	75	16.7		207	17.5	107	15.4	
	N3	188	8.2	31	6.9		221	18.7	145	20.9	
(y)pM stage	M0	2192	98.3	429	98.2	0.800	1049	93.9	616	94.2	0.812
	M1	37	1.7	8	1.8		68	6.1	38	5.8	
Resection	R0	2139	93.7	413	93.2	0.714	1032	88.6	595	87.9	0.654
	R +	144	6.3	30	6.8		133	11.4	82	12.1	
Response to neo-adjuvant treatment	None	231	12.9	55	16.3	0.239	187	36.1	108	37.9	0.065
	Partial	1214	67.6	219	64.8		272	52.5	159	55.8	
	Complete	352	19.6	64	18.9		59	11.4	18	6.3	
Postoperative complication	Yes	1350	56.7	273	57.2	0.845	496	38.1	252	32.9	0.017
	Leakage	444	18.7	76	15.9	0.333	98	7.5	39	5.1	0.031
	Pulmonary	686	28.8	144	30.2	0.553	205	15.7	83	10.8	0.002
	Cardiac	289	12.1	57	11.9	0.904	78	6.0	35	4.6	0.169
Re-intervention		505	21.4	103	21.6	0.907	209	16.2	91	11.9	0.008
Median ICU stay (IQR)		2	1–4	2	1–5	0.618	0	0–1	0	0–1	0.135
Median LOS (IQR)		12	9–19	13	9–19	0.026	9	7–14	9	7–13	0.970
Short-term mortality^a^		79	3.4	18	3.9	0.579	74	5.8	33	4.4	0.172

Percentages for the variables are calculated out of the total number of actual results available, excluding the missing values. Percentages may not add up to 100% due to rounding

*ICU* intensive-care unit, *IQR* interquartile range, *LOS* length of stay, *pN* pathological N stage, *pT* pathological T stage, *pM* pathological M stage

^a^Short-term mortality is the combined 30-day and in-hospital mortality

### Survival

**Fig. 3:**
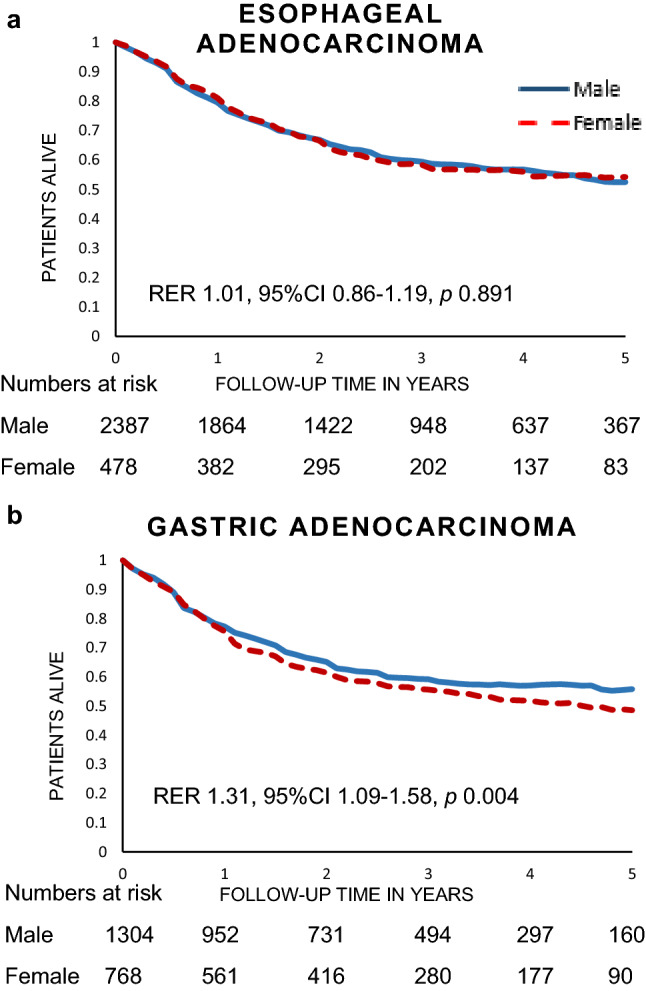
Relative survival of male and female patients with esophageal and gastric adenocarcinoma. **a** Relative survival of male and female patients with esophageal adenocarcinoma. **b** Relative survival of male and female patients with gastric adenocarcinoma. *RER* relative excess risk

For females with gastric cancer, 5-year relative survival was inferior to male patients (48.6% vs. 55.8%), also when adjusted for clinicopathological factors (RER 1.31, 95% CI 1.09–1.58, *p* = 0.004). No statistically significant differences in 5-year relative survival were observed between male and female patients with esophageal adenocarcinoma (52.4% vs. 54.2%; RER 1.01, 95% CI 0.86–1.19, *p* = 0.891; Fig. 3).

### Subgroup analysis

While the mean age at diagnosis was comparable between sexes, gastric cancer patients ≤ 55 years were more frequently of female sex (19.0% vs. 12.1%, *p* < 0.001). Young female gastric cancer patients were more frequently diagnosed with poor tumor differentiation (84.3% vs. 68.0%, *p* = 0.004), and diffuse-type tumors (72.5% vs. 50.4%, *p* = 0.004) compared to males (Online Resource 1). For older female gastric cancer patients, percentages of poor differentiation (66.0% vs. 56.5%, *p* < 0.001) and diffuse subtype (42.9% vs. 34.9%, *p* = 0.018) remained significantly higher compared to males, although the inter-sex difference was less pronounced.

For both female esophageal cancer patients ≤ 55 and > 55 years, relative survival was comparable to male patients (Online Resource 2), while for female gastric cancer patients, relative survival was inferior to males, especially for those ≤ 55 years (RER 1.41, 95% CI 1.03–1.94, *p* = 0.033).

## Discussion

While increasing evidence suggests an impact of the patients’ sex on the balance between efficacy and toxicity of systemic treatments in oncology [13, 14], its impact on surgical treatment choices and outcomes for gastric and esophageal cancers is largely undefined. In this context, our study reveals several major findings. In addition to sex differences in tumor location and histology of gastric cancer, a superior relative survival was observed in male patients, despite a higher postoperative morbidity rate. In contrast, while the use of neo-adjuvant treatment differs significantly between male and female esophageal cancer patients, postoperative morbidity and 5-year relative survival were comparable.

Specifically, in female gastric cancer patients, tumors were more often located in the antrum, while in male patients, tumors were more frequently located in the gastric fundus. No sex differences were observed in tumor location for esophageal adenocarcinoma, while in the literature, a more proximal esophageal tumor location has been described in female patients [28, 29]. In line with the previous studies, we observed more poorly differentiated and diffuse-type gastric cancers in female patients [30]. As much as histologic and molecular subtypes are distributed according to a characteristic pattern within the stomach and esophagus [31–33], these cancers seem to be distributed in a characteristic pattern for male and female patients, reflecting a “sexual dimorphism” most likely related to biological sex differences in cancer susceptibility and tumor biology [34].

Furthermore, significant differences in treatment allocation were observed in the current study. Even when adjusted for age, ASA-class, the presence of comorbidities, clinical stage, tumor differentiation, histopathological subtype, tumor location, and year of surgery, females with esophageal adenocarcinoma were significantly less frequently treated with neo-adjuvant therapy. No rational explanation for this treatment gap could be identified based on relevant clinicopathological factors, implicating that other factors must contribute. Potentially, unconscious gender bias in medical decisions, as described for other diseases, might play a role [35–38]. In addition, female patients less often received chemotherapy with concurrent radiotherapy as neo-adjuvant regimen. This observation is consistent with the recent study of Nobel et al. [28], in which they consider prior mediastinal irradiation for breast cancer as a possible explanation for the less frequent administration of concurrent radiotherapy. Within the DUCA registry, more females had a history of malignancy; however, as type of previous cancer and mediastinal irradiation is not registered, this hypothesis could not be confirmed. Although the previous studies on chemotherapy in gastric and esophageal cancer treatment observed more toxicity in female patients, resulting in less cycles of chemotherapy [14], the current study found no difference in the percentage of patients who completed neo-adjuvant therapy.

Only for patients with gastric cancer, postoperative morbidity and re-intervention rate differed between the sexes; with more complications and more re-interventions in male patients. The higher incidence of postoperative morbidity in male patients might be explained by a higher incidence of pre-operative comorbidity and a more extensive surgical procedure, with more males undergoing a total gastrectomy in the current study [39, 40]. Of interest, the higher incidence of anastomotic leakage in male patients with gastric cancer is also frequently observed after surgery of the lower gastro-intestinal tract, with male patients at higher risk for anastomotic leakage [41, 42].

Rates of incomplete tumor removal were comparable between sexes with gastric and esophageal cancer, although higher after gastric cancer surgery. While this is in line with the literature, incomplete tumor removal negatively impacts prognosis and is associated with low annual hospital volumes, emphasizing the need for further centralization of surgical gastric cancer care [43, 44].

Since life expectancy is known to differ between male and female patients, we chose to correct this by estimating and comparing the relative survival according to the method described by Pohar Perme [23]. No significant differences in 5-year relative survival were observed between male and female patients with esophageal adenocarcinoma, portraying similar survival of both sexes with esophageal cancer allocated to surgical treatment. While this comparable survival is in line with the previous studies [28, 29], they did not take differences in life expectancy into account.

In contrast, for gastric cancer, a superior relative survival was observed in male patients. Considering the prognostic favorable lower rate of comorbidities and postoperative morbidity [21], as well as the lower pathological T stages and more distal tumor locations observed in female patients [45, 46], the inferior survival of female gastric cancer patients might be explained by differences in tumor biology, such as the higher rate of poorly differentiated and diffuse-type tumors in females, which counterbalances the favorable impact of lower comorbidities, postoperative morbidity, pathological T stage, and tumor location. This observation is consistent with others; both Dutch [47] and Norwegian [48] population-based studies demonstrated higher proportions of diffuse-type gastric cancer in female patients and a significantly poorer survival for diffuse-type cancer. Furthermore, this observation does not seem to be limited to Caucasians; a large retrospective Korean study [30] confirms the higher rate of undifferentiated and diffuse-type gastric tumors in female patients and their negative prognostic impact.

Moreover, sex differences are potentially modulated by age [38] and might be the result of differences in exposure to sex hormones [6, 24, 25]. In the current study, especially young female gastric cancer patients were diagnosed with poor tumor differentiation and diffuse-type tumors, and showed inferior relative survival compared to males, which is consistent with the literature [30, 49]. Although a protective effect of female (pre-menopausal) sex hormones is hypothesized, the current study observed more female gastric cancer patients aged ≤ 55 years, with more prognostic negative tumor characteristics and a poorer relative survival. As a consequence of the observed sex differences in tumor biology and prognosis, sex-specific multimodal treatment strategies merit consideration and investigation in clinical trials.

There were some limitations to the present study. Although the validation of the matched DUCA-Vektis dataset was not published until November 2019 [21], the matching was already performed in September 2017. Since no additional update was performed, more recent data could not be included in this study. Another limitation to this combined dataset is the error margin up to 6%, caused by the incorrect assumption of death when health care insurance was terminated, e.g., in case of emigration [21]. However, there is no reason to assume that this error affected male and female patients differently and subsequently affected the results of our study. Due to the purpose of this registry, we were not able to include patients allocated to non-surgical treatment strategies, such as definitive chemoradiotherapy. Therefore, we cannot exclude that the observed differences might be due to a different allocation of male and female patients to surgical treatment. Additionally, due to the anonymous character of the DUCA dataset, we were not able to include other variables of interest, such as socio-economic status, which might affect access to care and treatment allocation, toxicity, or a possible discrepancy between treatment advised by the multidisciplinary team and actual treatment chosen.

Future research should include all patients diagnosed with gastric and esophageal cancer, regardless of treatment, to enable further investigation of sex differences in tumor characteristics, treatment allocation, and subsequent outcomes.

In conclusion, this study clearly demonstrates statistically significant and clinically relevant sex differences in tumor characteristics, treatment allocation, postoperative morbidity, and survival among surgically treated patients with gastric and esophageal cancer. As such, it provides an illustrative example of how sex and gender modulate surgical risks and outcomes. While the observed differences in gastric cancer histology are most likely related to biological sex differences in cancer susceptibility and tumor biology, also referred to as “sexual dimorphism” in cancer, differences in patient or tumor characteristics do not explain the observed treatment gap between male and female patients with esophageal adenocarcinoma, which might be attributable to other factors, such as unconscious gender bias. Consequently, the consideration of sex and gender differences in surgical research and treatment decisions is necessary and considered as an important step toward the individualization of gastric and esophageal cancer treatment.

## Supplementary Information

Below is the link to the electronic supplementary material.

Supplementary file1 (PDF 210 KB)

Supplementary file2 (PDF 149 KB)
