# Supplementary material for: Sex differences in tumor characteristics, treatment, and outcomes of gastric and esophageal cancer surgery: nationwide cohort data from the Dutch Upper GI Cancer Audit
Source: Gastric Cancer. 2021 Aug 7;25(1):22–32. doi: 10.1007/s10120-021-01225-1 (PMC8732809; doi:10.1007/s10120-021-01225-1)
Supplement: Supplementary file 1 — Supplementary file1 (PDF 210 KB) [file 10120_2021_1225_MOESM1_ESM.pdf]

Gastric Cancer

Sex Differences in Tumor Characteristics, Treatment and Outcomes of Gastric and Esophageal Cancer Surgery; Nationwide Cohort Data from the Dutch Upper-GI Cancer Audit

Authors: Marianne C Kalff, Anna D Wagner, Rob HA Verhoeven, Valery EPP Lemmens, Hanneke WM van Laarhoven, Suzanne S Gisbertz, Mark I van Berge Henegouwen, on behalf of the Dutch Upper GI Cancer Audit group

Affiliation: Department of Surgery, Cancer Center Amsterdam, Amsterdam UMC, University of Amsterdam, Amsterdam, The Netherlands.

m.i.vanbergehenegouwen@amsterdamumc.nl

Supplementary Table 1: Baseline tumor characteristics of male patients compared to female patients ≤55 years

		ESOPHAGEAL CANCER					GASTRIC CANCER				
		Male		Female ≤55 years			Male		Female ≤55 years		
		n = 2387		n = 75			n = 1304		n = 146		
Characteristics		n	%	n	%	*p*	n	%	n	%	*p*
cT stage	*Tis*	9	0.4	-	-	0.541	7	0.7	-	-	0.265
	T1	115	5.1	5	6.9		76	8.0	13	12.7	
	T2	445	19.6	19	26.4		252	26.6	20	19.6	
	T3	1644	72.2	46	63.9		541	57.0	62	60.8	
	T4	63	2.8	2	2.8		73	7.7	7	6.9	
cN stage	*N0*	829	36.0	32	43.8	0.172	648	58.1	73	59.8	0.715
	N+	1472	64.0	41	56.2		467	41.9	49	40.2	
Differentiation	*Good*	866	55.2	25	54.3	0.913	437	43.5	17	15.7	<0.001
	Poor	704	44.8	21	45.7		567	56.5	91	84.3	
Histological subtype	*Intestinal*	788	81.2	21	84.0	0.857	515	57.7	23	22.5	<0.001
	Diffuse	116	12.0	3	12.0		311	34.9	74	72.5	
	Mixed	66	6.8	1	4.0		66	7.4	5	4.9	
Clinical tumor location	*Proximal*	6	0.3	1	1.3	0.185					0.003
	Middle	81	3.4	4	5.3						
	Distal	1527	64.4	44	58.7						
	GEJ	757	31.9	26	34.7						
	Fundus						136	10.9	10	7.2	
	Corpus						389	31.2	40	29.0	
	Antrum						468	37.5	54	39.1	
	Pylorus						104	8.3	16	11.6	
	Entire stomach						73	5.8	17	12.3	
	Gastric remnant						78	6.3	1	0.7	

Percentages for the variables are calculated out of the total number of actual results available, excluding the missing values. Percentages may not add up to 100% due to rounding. cN clinical N stage, cT clinical T stage, GEJ gastro-esophageal junction.

Supplementary Table 2: Baseline tumor characteristics of male patients compared to female patients >55 years

		ESOPHAGEAL CANCER					GASTRIC CANCER				
		Male		Female >55 years			Male		Female >55 years		
		n = 2387		n = 75			n = 1304		n = 146		
Characteristics		n	%	n	%	*p*	n	%	n	%	*p*
cT stage	*Tis*	9	0.4	-	-	0.069	7	0.7	3	0.7	0.474
	T1	115	5.1	32	8.3		76	8.0	44	9.9	
	T2	445	19.6	67	17.4		252	26.6	113	25.3	
	T3	1644	72.2	274	71.4		541	57.0	242	54.3	
	T4	63	2.8	11	2.9		73	7.7	44	9.9	
cN stage	*N0*	829	36.0	162	42.1	0.023	648	58.1	304	57.0	0.715
	N+	1472	64.0	223	57.9		467	41.9	229	43.0	
Differentiation	*Good*	866	55.2	138	51.5	0.265	437	43.5	161	34.0	<0.001
	Poor	704	44.8	130	48.5		567	56.5	313	66.0	
Histological subtype	*Intestinal*	788	81.2	139	81.8	0.439	515	57.7	216	50.3	0.018
	Diffuse	116	12.0	16	9.4		311	34.9	184	42.9	
	Mixed	66	6.8	15	8.8		66	7.4	29	6.8	
Clinical tumor location	*Proximal*	6	0.3	1	0.2	0.886					<0.001
	Middle	81	3.4	17	4.2						
	Distal	1527	64.4	257	64.1						
	GEJ	757	31.9	126	31.4						
	Fundus						136	10.9	27	4.5	
	Corpus						389	31.2	192	32.3	
	Antrum						468	37.5	288	48.5	
	Pylorus						104	8.3	48	8.1	
	Entire stomach						73	5.8	35	5.9	
	Gastric remnant						78	6.3	4	0.7	

Percentages for the variables are calculated out of the total number of actual results available, excluding the missing values. Percentages may not add up to 100% due to rounding. cN clinical N stage, cT clinical T stage, GEJ gastro-esophageal junction.
