# Supplementary material for: Sex differences in tumor characteristics, treatment, and outcomes of gastric and esophageal cancer surgery: nationwide cohort data from the Dutch Upper GI Cancer Audit
Source: Gastric Cancer. 2021 Aug 7;25(1):22–32. doi: 10.1007/s10120-021-01225-1 (PMC8732809; doi:10.1007/s10120-021-01225-1)
Supplement: Supplementary file 2 — Supplementary file2 (PDF 149 KB) [file 10120_2021_1225_MOESM2_ESM.pdf]

Sex Differences in Tumor Characteristics, Treatment and Outcomes of Gastric and Esophageal Cancer Surgery; Nationwide Cohort Data from the Dutch Upper-GI Cancer Audit

Gastric Cancer

Marianne C Kalff, Anna D Wagner, Rob HA Verhoeven, Valery EPP Lemmens, Hanneke WM van Laarhoven, Suzanne S Gisbertz, Mark I van Berge Henegouwen, on behalf of the Dutch Upper GI Cancer Audit group

Department of Surgery, Cancer Center Amsterdam, Amsterdam UMC, University of Amsterdam, Amsterdam, The Netherlands.

m.i.vanbergehenegouwen@amsterdamumc.nl

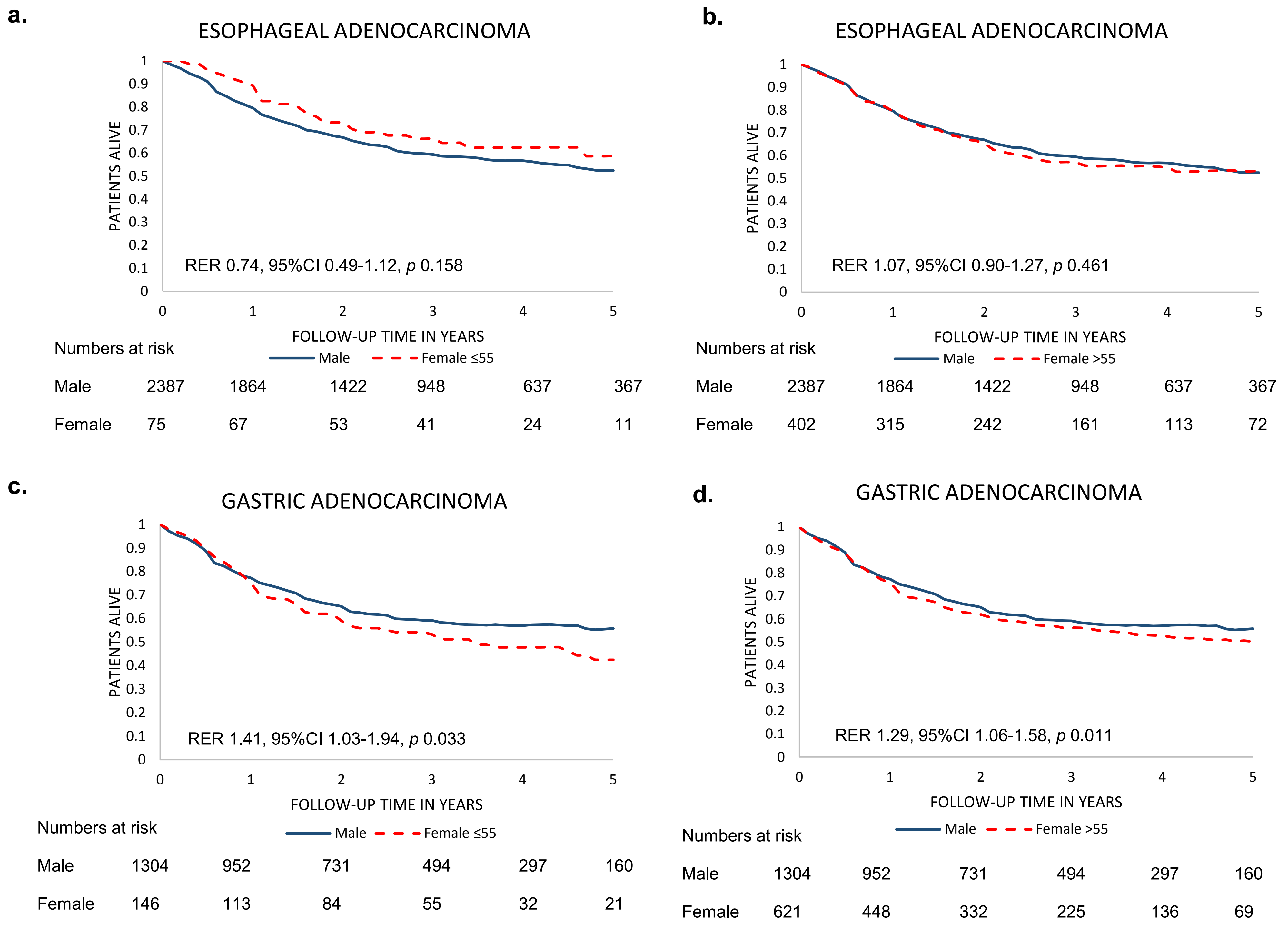

a. Relative survival of male patients and female patients ≤55 years with esophageal adenocarcinoma. **b.** Relative survival of male patients and female patients >55 years with esophageal adenocarcinoma. **c.** Relative survival of male patients and female patients ≤55 years with gastric adenocarcinoma. **d.** Relative survival of male patients and female patients >55 years with gastric adenocarcinoma.
